# Counting and Number Line Trainings in Kindergarten: Effects on Arithmetic Performance and Number Sense

**DOI:** 10.3389/fpsyg.2018.00975

**Published:** 2018-06-19

**Authors:** Ilona Friso-van den Bos, Evelyn H. Kroesbergen, Johannes E. H. Van Luit

**Affiliations:** ^1^Department of Special Education, Cognitive & Motor Disabilities, Utrecht University, Utrecht, Netherlands; ^2^Behavioural Science Institute, Radboud University, Nijmegen, Netherlands

**Keywords:** number sense, counting, number line, training, children, arithmetic

## Abstract

Children’s early numerical capacities form the building blocks for later arithmetic proficiency. Linear number placements and counting skills are indicative of mapping, as an important precursor to arithmetic skills, and have been suggested to be of vital importance to arithmetic development. The current study investigated whether fostering mapping skills is more efficient through a counting or a number line training program. Effects of both programs were compared through a quasi-experimental design, and moderation effects of age and socio-economic status (SES) were investigated. Ninety kindergartners were divided into three conditions: a counting, a number line, and a control condition. Pretests and posttests included an arithmetic (addition) task and a battery of number sense tasks (comparison, number lines, and counting). Results showed significantly greater gains in arithmetic, counting, and symbolic number lines in the counting training group than in the control group. The number line training group did not make significantly greater gains than the control group. Training gains were moderated by age, but not SES. We concluded that counting training improved numerical capacities effectively, whereas no such improvements could be found for the number line training. This suggests that only a counting approach is effective for fostering number sense and early arithmetic skills in kindergarten. Future research should elaborate on the parameters of training programs and the consequences of variation in these parameters.

## Introduction

Children’s early numerical capacities have received growing interest in the past decade: numerical skills in kindergarten form the building blocks for later proficiency in mathematics (e.g., Passolunghi and Lanfranchi, 2012; Hornung et al., 2014). Number sense is the term most often used to characterize the intuitive understanding of number and quantities and their relations (Dehaene, 1997; Gersten and Chard, 1999; Spelke, 2000). Number sense refers to a cognitive framework that allows a child to understand, for example, the difference between having two or three sweets, but gradually develops into a much more advanced system of conceptual knowledge that allows a person to intuitively understand abstract number relations and algorithms. Various skills have been thought to be at the root of number sense development, among which the ability to map between symbolic and non-symbolic magnitudes (Dehaene, 2001; Mundy and Gilmore, 2009; Desoete et al., 2012; Kolkman et al., 2013). In the current study, this mapping ability is trained in typically performing kindergartners using two different training programs, in order to investigate how the skill is best fostered and how arithmetic skills can be fostered through mapping.

Kindergarten number sense can be divided into three skills: non-symbolic skills, symbolic skills, and mapping between non-symbolic and symbolic skills. Mapping has been found to be the strongest predictor of later mathematical performance, and was hypothesized to restructure non-symbolic number knowledge of a child into a more conventional cognitive concept of mathematics (Kolkman et al., 2013). Mapping is considered to lie at the root of adequate development of arithmetic skills (Siegler and Booth, 2004; Booth and Siegler, 2008; Wong et al., 2016) and refers to a flexible integration between non-symbolic and symbolic quantity processing, meaning that children with well-developed mapping skills are able easily to transcode between number words, number symbols, and non-symbolic quantities. This transcoding ability may also make symbolic quantities more meaningful to children, which is essential for adequate arithmetic development (Wong et al., 2016).

Two lines of enquiry focus on the formation of mapping skills in young children, the first of which focuses on counting skills. Counting is considered a prerequisite for forming links between symbolic and non-symbolic processing (Lipton and Spelke, 2005; Le Corre and Carey, 2007). Reciting the counting sequence may help children understand the cardinal value of number words, thereby realizing that each number word relates to an exact quantity using bottom-up processing (Noël and Rousselle, 2011). In bottom-up processing, the individual stimulus, in this case the quantity or number, is used to construct an understanding of a system as a whole, in this case a system of numbers and their quantitative relations such as bigger and smaller numbers. A second line of enquiry focuses on linear placements of numbers on a number line, which are indicative of mapping skills. Acuity on a number line task is predictive of mathematics performance (Booth and Siegler, 2008; Schneider et al., 2018), and can be fostered in a top-down processing framework through number line activities, such as playing numerical linear board games (Siegler and Ramani, 2009; Fisher et al., 2011; Dackermann et al., 2016), thereby forming a novel approach to intervention in numerical skills. In this top-down presentation of number relations, numbers are understood through their placement on a scale with a predetermined number range, which forms the context for the task, and the scale itself must be understood before individual items can be placed on the scale (the number line).

### Counting

Knowledge of counting and number symbols is considered an important predictor of arithmetic performance (Kolkman et al., 2013). Counting skills could predict the extent to which children can estimate numerosities (Lipton and Spelke, 2005) and place numbers on an empty number line (Desoete et al., 2013; Simms et al., 2013; Friso-van den Bos et al., 2014). It has been proposed that finger counting helps children associate between symbolic magnitudes and non-symbolic sets of items through the finger pattern, as well as understand basic operations such as addition (Noël, 2005; Gracia-Bafalluy and Noël, 2008; Moeller et al., 2011) using a bottom-up process in which combining small numbers of objects into a bigger unit developmentally precedes more complex operations with bigger numbers. Nearly all number sense trainings include practicing counting procedures and knowledge of number symbols (e.g., Van Luit and Schopman, 2000; Krajewski et al., 2008; Kroesbergen et al., 2012; Toll and Van Luit, 2014), and isolated practice of counting procedures has been found to generalize to improved multiplication proficiency (Blöte et al., 2006). It was suggested that mapping, as the most important factor of number sense, develops through counting skills, as described by Le Corre and Carey (2007), who postulated that children make analogies between the sequence in the count list and quantifiable sets of objects, and use induction to learn to understand the correspondence between the addition of an item to a set and the progression through the count list. This implies that the mapping between number words and tangible quantities is first understood by a child through the bottom-up process of counting, making counting a first step toward a more abstract concept of number.

### Number Lines

Number line placement acuity is also thought important for the development of both arithmetic skill and broader mathematical skills (Geary et al., 2007; Booth and Siegler, 2008; Schneider et al., 2018). In a number line task, a child places a target number on an empty number line bounded by the begin- and endpoints marked on either side of the line – a top-down approach in which the number range has been framed and individual units need to be placed within this framework. To use number lines in number tasks, children need to be able to relate a number to the corresponding quantity and consequently realize that a number obtains its position on the number line through its quantitative value. Typically, young children make non-linear placements, adhering to a logarithmic or power model of placements, while older children show a more linear pattern of number placement, with equal spacing between numbers of various sizes (Siegler and Opfer, 2003; Siegler and Booth, 2004; Booth and Siegler, 2006; Barth and Paladino, 2011). A more linear pattern of placements is predictive of higher achievement in arithmetic in children (Booth and Siegler, 2008). Acuity of number line placements may be interpreted as a child’s ability to map between symbolic numbers and non-symbolic quantities (Kolkman et al., 2013). The symbolic numbers, in this interpretation, are the numbers to be placed on the empty number line, and the non-symbolic quantity is represented as the continuous space between the extremities of the number line.

The training of number line placement has received growing interest in the past few years (e.g., Fisher et al., 2011; Ramani and Siegler, 2011; Dackermann et al., 2016). Playing numerical linear board games, in which linear ordering of numbers was emphasized, has repeatedly been reported to improve successfully number line acuity (Siegler and Ramani, 2009; Fisher et al., 2011) and thereby facilitate the mapping between numbers and quantities. Furthermore, placement of numbers along the continuum of a number line may be seen as a form of visual imagery of number information; a prerequisite for successful acquisition of the more complex algorithm skills needed in advanced mathematics (Zhang et al., 2012). Number line training has also been demonstrated to enhance arithmetic performance in a study among kindergarten children, but there was no transfer effect to other measures of number sense (Maertens et al., 2016).

### The Current Study

The present study aimed to investigate whether the development of number sense and consequent arithmetic skills is more efficient through a bottom-up counting or a top-down number line training program in comparison to a control group through experimental training studies. We expected both trainings to have significant effects on measures of arithmetic and mapping, and small transfer effects on symbolic number sense measures. Moreover, we expected children enrolled in a number line training to make greater gains on a measure of number line acuity and children enrolled in a counting training to make greater gains on a counting task than other groups, because these skills were directly trained in these groups. However, we did not expect significant training gains on non-symbolic number sense measures because previous research showed no direct relations between non-symbolic number sense and mapping, and relations between arithmetic and number sense were dominated by mapping skills rather than non-symbolic number sense (Kolkman et al., 2013). Measures of non-symbolic number sense were included nevertheless to get a full account of training effects on number sense. Gains made after the interventions may reflect the way in which kindergarten children normally (without intervention) construct number knowledge, because according to Piaget’s theory of cognitive development (Piaget, 1970), an intervention that fosters knowledge the way children intuitively approach it is likely to have greater effect than an intervention that teaches children to think differently about the matter at hand than they intuitively do.

Intervention in number sense skills in children of low socio-economic status (SES) has aroused specific research interest (Siegler and Ramani, 2008; Jordan et al., 2012; Dyson et al., 2013). Greater gains have been reported for children from a low SES background than for children from middle SES backgrounds (Starkey et al., 2004). The current study attempted to replicate this finding by creating a distinction between children from high or low to average SES and investigating whether training is equally effective for both groups of children. Finally, because age has been found to explain differences in intervention outcomes between studies (Kroesbergen and Van Luit, 2003), the age of the children was included as a moderator variable.

## Materials and Methods

### Participants

Ninety Dutch kindergartners with a mean age of 5 years and 8 months (*SD* = 4 months; range: 5;0–6;6 years) participated in the study. Data of one child were removed because his data produced outliers on multiple variables. Of the remaining children, 47 were girls (52.8%). The number of children born in the Netherlands was 83 (93.2%). Of all children born outside of the Netherlands, at least one parent was born in the Netherlands.

In each class, nine children participated. After pretesting, children were divided into three conditions: (1) a counting training condition, (2) a number line training condition, and (3) an ‘education as usual’ control group. They were distributed across conditions in such a way that their counting and arithmetic scores at pretest were approximately equal between groups, with three children from each class participating in each condition, to regulate group size and prevent differences in key outcome measures at pretest. There was no age difference between the three groups, *F*(2,86) = 0.55, *p* = 0.58, nor was there a difference between any of the groups in any of the outcome measures during pretest (*p*s ranging from 0.44–0.97).

Socio-economic status was measured using short questionnaires filled out by parents. Children were coded as coming from families with high SES if they had at least one parent who had completed higher education. Of the children, 56 were coded as being from high SES families, 32 as being from low to average SES families, and for one child no data were available. Children from both SES categories were distributed equally across training conditions, χ^2^(2, *N* = 88) = 0.22, *p* = 0.89.

### Interventions

Each intervention group received 12 training sessions spread over 6 weeks in groups of three children, lasting approximately 20 min per session. Difficulty of the sessions increased by extending the range of numbers included in the games: numbers up to 10 were included in the first four sessions, numbers up to 20 in the next four sessions, and numbers up to 50 in the last four sessions. This range was especially included because most children at the end of kindergarten already know the range up to 20. In both training programs, four games were played in total, and two per session, so that each game was played every other session. Number cards were used to support the activities in each training program. Training sessions were not specifically planned during class mathematics activities.

#### Counting Training

The counting training consisted of the following activities, presented as games:

(1) Resultative counting, using various simple motor activities such as clapping, colorful cards, and objects to count. Counting skills are predictive of proficiency in other number-related tasks (Lipton and Spelke, 2005).
(1) Counting on from a number higher than 1. Various sets of colorful cards were used for this activity. Counting on is a counting activity that requires the understanding of cardinality (Van Luit and Van de Rijt, 2009).
(1) Non-linear board game: in this board game, a six-sided die was used to indicate the moves the children could make. The squares on the board were not numbered, and some of them contained an icon initiating a counting challenge. Playing non-linear board games is not expected to facilitate number line acuity, as opposed to linear board games (Ramani and Siegler, 2011).
(1) Counting on: colorful stones of the same shape and size were added to a pillow case to stimulate the use of shortened counting and illustrate the concept of addition.

#### Number Line Training

The number line training consisted of the following activities, presented as games:

(1) Number-to-position game: an empty number line in the form of a printed test tube was presented on worksheets, and a number was located on the number line by the children with a pencil. This game was based on the number-to-position task (Siegler and Booth, 2004; Laski and Siegler, 2007). For each session, ten numbers were chosen that covered the entire range of the number line, and the order of the items was semi-randomized.
(1) Position-to-number game: a number line with a given position was presented on worksheets, and the children were asked to assign a number to the location. This activity was based on the number line task (Siegler and Booth, 2004; Laski and Siegler, 2007), but the position was the information given to the child, and the number the response by the child. The beginning and endpoint were given. Target numbers covered the entire range of the number line and were presented in semi-random order.
(1) Linear numerical board game (Siegler and Ramani, 2008): in this board game, a six-sided die was used to indicate the moves the children could make. The squares on the board were numbered, and the child was asked to state out loud which squares were crossed during a move.
(1) Linear numerical tag game: in this game, children simulated a tag game on a board with numbered squares. They chose a starting position and took turns to roll a die indicating the number of steps (one to six) they could move in either direction of the number line. They were challenged to try to locate their tokens on the same square as another child and received points for each time this happened. The children were asked to state out loud which squares were crossed during their move.

#### Control Group

A control group received education as usual and did not participate in any research-related extra activities. Children in the Netherlands typically receive full-day programs from the day they turn 4 years old. Mathematics is part of every kindergarten curriculum, and is taught through various age-appropriate activities.

### Measures

#### Arithmetic

To measure early arithmetic proficiency, children completed 16 addition problems displayed on the laptop screen. Of the problems, 11 had an answer below 10 (e.g., 5 + 3) and 5 had an answer between 10 and 20 (e.g., 6 + 8). Tie problems were not included in the set of items. All problems were preceded by a 2-s alerting phase. The score was the number of correct answers. Internal consistency at pretest was high, α = 0.94.

#### Number Sense: Comparison Tasks

The comparison task had two versions. In the symbolic version, participants judged which of two Arabic numbers was bigger than the other through a key press using the hand corresponding to the location (left or right) of the selected stimulus on the screen. All numbers were between 1 and 9. Each trial was preceded by an alerting beep, and 1500 ms after the beep, the stimuli appeared. The maximum response time was 5 s. There were four practice trials and 26 test trials, and total accuracy was used as the score of the child. Numerical distance could range from 1 to 4, each distance appearing 8, 7, 6, and 5 times, respectively. The largest number appeared on both sides of the screen 13 times. Symbolic comparison tasks can be seen as measures of mapping because it has been hypothesized that children use the mental number line to complete the task (see: Kolkman et al., 2013). Test–retest reliability of a similar task has been found to be good (Clarke and Shinn, 2004).

The non-symbolic version of the comparison task was mostly the same as the symbolic version, but sets of dots appeared instead of Arabic numbers, controlled for dot size and surface array, and counterbalanced for the location of the correct response. To prevent counting strategies, the stimuli disappeared from the screen after 840 ms. Numerical distance could range from 1 to 4, each distance appearing 8, 7, 6, and 5 times, respectively, with a total of 26 trials. All trials were preceded by an alerting beep, and 1500 ms after the beep, the stimuli appeared. The maximum response time was 5 s. There were four practice trials and 26 test trials, and total accuracy was used as the score of the child. Numerical distance could range from 1 to 4. Non-symbolic comparison tasks can be seen as measures of non-symbolic NS.

#### Number Sense: Number Line Tasks

The number line task (Siegler and Opfer, 2003) had two versions. In the symbolic version, Arabic numbers between 1 and 100 were displayed onscreen below a horizontal line. On both sides of the line, the minimum (1) and maximum (100) were given, and participants pointed to the position on the line they selected for the target number. Twenty-two test trials were presented to the participants, preceded by two practice trials in which they located the numbers 1 and 100 on the line and received feedback. Test trials were the numbers 2, 4, 9, 11, 14, 17, 23, 26, 31, 38, 44, 45, 52, 59, 61, 66, 73, 78, 84, 86, 92, and 99. Trials were presented in random order. No feedback was given during the testing phase. Symbolic number line tasks can be seen as measures of mapping (Kolkman et al., 2013). The score of the child was the explained variance of a linear slope (*R*^2^), indexed by the squared correlation between estimated and actual positions.

The non-symbolic version of the number line task was similar to the symbolic version, the only difference being that the children located arrays of dots on the number line. We did not control for any visual properties of these dots such as size or surface array. Minimum and maximum were displayed in non-symbolic form as well. The same numbers were used as in the symbolic version, and numbers were also presented in random order. The score of the child was the explained variance of a linear slope (*R*^2^). Non-symbolic number line tasks can be seen as measures of non-symbolic NS.

#### Number Sense: Counting

To measure the counting skills, subscales of the ENT-R (Van Luit and Van de Rijt, 2009) were used. The original ENT-R consists of nine subscales. In this study, only the subscales that measure counting were used, namely: (1) Use of number words, such as rote counting; (2) Structured counting, such as counting in two’s; (3) Resultative counting, such as counting out a set of objects; and (4) General understanding of number words, such as indicating which whole number is exactly between two other numbers. Each subscale contains five items with counting tasks ranging up to 20. Resultative counting up to 20 is expected of children at the end of kindergarten. One point was awarded for each correct answer. Internal consistency of this test is good (Van Luit and Van de Rijt, 2009). This scale can be seen as a measure of symbolic number sense (Kolkman et al., 2013).

### Procedure

The current study was part of the MathChild study, which was funded with a project grant from the Netherlands Organisation for Scientific Research (NWO), grant 411-07-113. The study proposal was evaluated for both quality and ethical standards, and approved by NWO. The study conformed to national and international standards of ethical research, as summarized in the Netherlands Code of Conduct for Academic Practice (Association for Universities in the Netherlands, 2004). Active parental consent was obtained prior to data collection.

Pretests and posttests were conducted individually with an interval of 6–8 weeks. The children were tested in a quiet room inside the school by undergraduate students. All tasks were administered on a laptop computer using E-Prime 1.2 software (Psychological Software Tools^1^). Because of budget limitations, a variety of laptop computer brands and models was used, therefore screen sizes varied as well. Prior to testing, the students administering the tests were trained in the use of the software and the standardized instruction and registration of the tasks in a 2-h group session and successive self-guided practice exercises covering all the instruments. The pretest and posttest tasks were divided into two sessions, which took place on 2 days no more than 1 week apart. After each session, children were rewarded with a colorful sticker. During the first session, children completed working memory tasks (not included in the current analyses), arithmetic, symbolic and non-symbolic comparison, and during the second session, children completed the ENT, the symbolic number line task, and the non-symbolic number line task. No variations in task order were made.

Training sessions took place inside the school, in groups of three children, and were led by trained undergraduate students. Sessions were planned with the teacher and conducted by the undergraduate students. The training sessions were not digitalised, but conducted using colorful materials such as play boards and pawns. Posttesting took place no more than 2 weeks after the last session.

### Analytical Strategy

To address the research questions, Hierarchical Linear Modeling (HLM) was applied using the software package HLM version 6.06. Scores on the various tasks measuring arithmetic proficiency and number sense served as dependent variables. Three-level hierarchical models were estimated with measurement occasion at the first level, individual children at the second level, and the groups in which children were trained at the third level (children in the control group were nested in kindergarten classes). Main effects of occasion (level 1) and training condition (level 3) were added first, and interactions between occasion and training condition were added in a second step, indicating differential growth of children in each of the conditions. If significant interactions were found, *post hoc* analyses using the counting training as a reference category were conducted to investigate the difference between children in the counting and number line condition.

To control for Type I errors, the Benjamini–Hochberg correction was used, in which alpha values are adjusted for the number of analyses reported (in this case: six hierarchical regression analyses, or one analysis for each of the measures listed) based on the rank-order of *p*-values (Benjamini and Hochberg, 1995). Probability values are not compared with a static alpha value, but with a corrected ‘α. Separate corrections were performed for *post hoc* analyses.

## Results

Correlations between measures at pretest and moderators can be found in **Table 1**. Descriptive statistics of all three groups and the total sample in each measure can be found in **Table 2**.

**Table 1:** Correlations between measures at pretest, and moderator variables.

	1	2	3	4	5	6	7
(1) Symbolic comparison	–						
(2) Non-symbolic comparison	0.31^∗∗^	–					
(3) Symbolic number line	0.33^∗∗^	0.34^∗∗^	–				
(4) Non-symbolic number line	0.23^∗^	0.36^∗∗^	0.45^∗∗∗^	–			
(5) Counting	0.38^∗∗∗^	0.37^∗∗∗^	0.58^∗∗∗^	0.47^∗∗∗^	–		
(6) Arithmetic	0.42^∗∗∗^	0.37^∗∗∗^	0.58^∗∗∗^	0.51^∗∗∗^	0.72^∗∗∗^	–	
(7) Age	0.03	0.13	0.12	0.18	0.23^∗^	0.25^∗^	–
(8) SES	0.11	0.22^∗^	0.05	0.02	0.22^∗^	0.19	


**^∗^p < 0.05, ^∗∗^p < 0.01, ^∗∗∗^p < 0.001; all correlations are Pearson’s correlations, except correlations with SES, which are Spearman’s rank correlations.**

**Table 2:** Descriptive statistics at pretest and posttest, for the counting group, number line group, control group, and total sample.

Task	Counting group		Number line group		Control group		Total	
				
	Pretest mean (*SD*)	Posttest mean (*SD*)	Pretest mean (*SD*)	Posttest mean (*SD*)	Pretest mean (*SD*)	Posttest mean (*SD*)	Pretest mean (*SD*)	Posttest mean (*SD*)
Arithmetic	6.03 (5.67)	7.93 (4.47)	5.93 (5.46)	6.77 (5.42)	6.85 (5.54)	7.14 (4.97)	6.25 (5.51)	7.27 (4.94)
Symbolic comparison	20.67 (4.77)	20.97 (4.49)	20.77 (5.06)	21.83 (3.99)	21.28 (4.41)	22.10 (2.80)	20.90 (4.71)	21.63 (3.82)
Non-symbolic comparison	20.83 (4.46)	21.43 (3.76)	20.93 (4.70)	20.48 (3.92)	21.10 (3.50)	21.52 (3.44)	20.96 (4.21)	21.14 (3.70)
Symbolic number line^∗^	0.32 (0.25)	0.47 (0.23)	0.35 (0.26)	0.45 (0.29)	0.40 (0.27)	0.41 (0.30)	0.36 (0.26)	0.44 (0.27)
Non-symbolic number line	0.55 (0.22)	0.66 (0.20)	0.54 (0.26)	0.65 (0.22)	0.48 (0.28)	0.63 (0.27)	0.52 (0.25)	0.65 (0.23)
Counting (ENT-R)	11.13 (4.98)	14.43 (4.01)	11.50 (4.69)	13.83 (4.29)	11.79 (4.04)	13.55 (4.14)	11.47 (4.55)	13.94 (4.12)


**^∗^Scores on the number line tasks reflect fit with a linear trend of individual data points. When using median estimates of all children on the symbolic number line task, fit with a linear and a logarithmic trend at pretest was comparable to previously reported estimates of fit (Berteletti et al., 2010): R^*2*^lin = 0.74, R^*2*^log = 0.96. Moreover, data of all except two children correlated positively with the presented numbers, indicating that the children understood the task well.**

### Training Effects

Results of the hierarchical regression analyses are reported in **Table 3** for all measures. Each model concerns one of the outcome measures. The variable Time is indicative of mean growth across all conditions between pretest and posttest. Analyses show that mean growth between pretest and posttest was significant for counting and for non-symbolic number line performance, but not for any other measure (**Table 3**).

**Table 3:** Hierarchical linear regression models for task performance.

	Arithmetic					Symb. comparison					Non-symb. comparison				
			
	*B*	β	*df*	*p*	‘α	*B*	β	*df*	*p*	‘α	*B*	β	*df*	*p*	‘α
Model intercept	6.88	-	27	<0.001***	-	21.02	-	27	<0.001***	-	20.96	-	27	<0.001***	-
Time	0.08	0.01	166	0.89	-	0.93	0.11	168	0.26	-	0.42	0.05	165	0.41	-
Count training	-0.85	-0.08	27	0.58	-	-0.36	-0.04	27	0.78	-	-0.13	-0.02	27	0.88	-
NL training	-0.79	-0.07	27	0.61	-	-0.23	-0.03	27	0.86	-	0.07	0.01	27	0.95	-
Time × count training	2.04	0.15	166	<0.01**	0.02	-0.62	-0.05	168	0.58	0.08	0.18	0.02	165	0.84	0.09
Time × NL training	0.78	0.06	166	0.34	0.07	0.17	0.01	168	0.88	0.10	-0.87	-0.08	165	0.17	0.04
	
	**Symb. number lines**					**Non-symb. number lines**					**Counting (ENT-R)**				
			
Model intercept	0.40	-	27	<0.001***	-	0.47	-	27	<0.001***	-	11.64	-	27	<0.001***	-
Time	-0.01	-0.01	166	0.89	-	0.16	0.32	165	<0.001***	-	1.61	0.18	168	<0.01**	-
Count training	-0.08	-0.14	27	0.32	-	0.07	0.13	27	0.33	-	-0.50	-0.01	27	0.74	-
NL training	-0.05	-0.09	27	0.55	-	0.07	0.13	27	0.34	-	-0.27	-0.03	27	0.86	-
Time × count training	0.16	0.23	166	<0.01**	0.01	-0.05	-0.08	165	0.30	0.06	1.69	0.14	168	0.03*	0.03
Time × NL training	0.10	0.14	166	0.08	0.03	-0.05	-0.08	165	0.31	0.06	0.88	0.07	168	0.25	0.05


**The control group served as a reference group in the presented analyses. NL, number line; Non-symb., non-symbolic; B, unstandardized coefficient; β, standardized coefficient. ‘α, adjusted alpha after Benjamini–Hochberg correction. ^∗^p < 0.05, ^∗∗^p < 0.01, ^∗∗∗^p < 0.001.**

The interactions between time and condition are indicative of divergence in growth between the experimental group and the control group, the latter group serving as a reference group. Results show that arithmetic scores were significantly predicted by an interaction between counting training and time, indicative of larger gains within the counting group (explained variance at the occasion level: 17.85%; see **Table 3**). There was no evidence for greater gains within the number line group than in the control group. *Post hoc* tests indicated that the counting group did not make greater progress than the number line group, *B* = -1.26, β = -0.09, *p* = 0.11.

There was no interaction between training condition and time on the symbolic comparison test (explained variance at the occasion-level: 3.66%; see **Table 3**). Interaction effects on scores of non-symbolic comparison were not significant either (explained variance at the occasion level: 2.56%). No *post hoc* analyses were conducted for either symbolic or non-symbolic comparison.

There was a significant interaction between training condition and time predicting scores on the symbolic number line test, indicative of larger gains in the group following the counting training in comparison to the control group, but not the number line training (explained variance at the occasion level: 21.50%; see **Table 3**). *Post hoc* analyses indicated that the counting group did not make more gains than the number line group, *B* = -0.07, β = -0.09, *p* = 0.25. There was no significant effect of an interaction between training condition and time on non-symbolic number line performance (explained variance at the occasion level: 34.68%; see **Table 3**).

Finally, in the model predicting counting (ENT-R) scores, there was a significant interaction between counting training and time (**Table 3**), but not between number line training and time, indicative of greater gains within the counting group, but not the number line group, than in the control group (explained variance at the occasion level: 45.43%). *Post hoc* tests indicated that the counting group did not progress more than the number line group *B* = -0.81, β = -0.06, *p* = 0.45.

### Moderation of SES and Age

For all measures in which there was divergence in growth between the experimental groups and the control group, main effects of SES and age were included, as well as interactions between these variables and training gains, to investigate whether change in scores in number sense and mathematics could be explained by variation in SES and/or age of the children. Significant interaction effects were indicative of divergence in growth between children of various SES or ages.

The SES did not predict growth in any of the measures for the children enrolled in the counting training, or for arithmetic, symbolic number line, or counting scores for children enrolled in either of the training groups (all *p*s > 0.05). Age of the children predicted growth of children enrolled in the counting training in arithmetic, *B* = -0.74, β = -4.57, *p* = 0.03, and symbolic number line scores, *B* = -0.04, β = -4.78, *p* < 0.01, but not in counting, *B* = -0.45, β = -3.21, *p* = 0.07, and it did not predict growth in scores of children enrolled in the number line training, all *p*s > 0.05.

## Conclusion and Discussion

In the current study, the possibilities of advancing number sense and arithmetic using bottom-up counting training and top-down number line training were investigated. Both counting skills and number line skills may be used to fine-tune mapping between symbolic and non-symbolic representations (Le Corre and Carey, 2007; Booth and Siegler, 2008; Noël and Rousselle, 2011), which may form a foundation for arithmetic development (Wong et al., 2016). The current study investigated mapping in a quasi-experimental design. We attempted to foster mapping skills using counting activities and number line activities, both of which have been hypothesized to advance mapping capacities in young children (Lipton and Spelke, 2005; Le Corre and Carey, 2007; Siegler and Ramani, 2009; Fisher et al., 2011).

Results indicate that kindergartners outperformed the control group only after counting training. This implies that number processing and consequent arithmetic skills can be nurtured through counting activities (Lipton and Spelke, 2005; Le Corre and Carey, 2007), and it may suggest that development in the school context is also furthered by counting more than by number line training. Formation of mapping skills may occur through the repeated bottom-up process of matching number words with visible quantities, as suggested by Le Corre and Carey (2007), and quantities may more easily be processed by assessing individual items in a set one-by-one than by placement in a higher-order framework, which is done in number line tasks using top-down processing. This more fluent processing may have led to significant gains in the counting group and not the number line group in comparison to the control group, even when measuring progress through a number line task. This is congruent with the notion that mapping fosters arithmetic skills by making symbolic numbers more meaningful to children (Wong et al., 2016); something that is likely more easily achieved through a tangible and observable counting process than through an abstract number line game. It should be noted though that differences in gains between the counting group and the number line group were not significant. Rather, the number line group made small (non-significant) progress on several tasks that imply that with sufficient power, number line activities would in fact show small effects on number sense measures, although not in the same order of magnitude as the counting training, nor would they be of the same order as the effects previously reported (e.g., Siegler and Ramani, 2009; Maertens et al., 2016).

Advances in mapping could be seen in the number line task, measuring mapping, but not on the symbolic comparison task, scores on which can also be seen as an indication of mapping skills (Kolkman et al., 2013). This may be due to the low difficulty of the task. Numbers ranged up to 9, and children showed no obvious difficulties completing the task. This is also apparent from their scores at pretest, during which children performed well above the chance level of 50%. Possibly, the few mistakes made by children were due to other factors such as attentional resources, rather than their mapping capacities.

It is also worth noting that the counting training had no effects on measures of non-symbolic processing. Effects of number sense training on non-symbolic tasks have previously been found to be lacking (Malofeeva et al., 2004) or to have smaller effects than on symbolic tasks (Wilson et al., 2006), suggesting that it is primarily the symbolic skill level that interacts with broader numerical development and plays a key-role in the development of number sense. It has been hypothesized that non-symbolic skills serve as a foundation for all further development in mathematics and number skills (Dehaene, 2001), but the current study suggests that limited gains in non-symbolic skills do not constrain gains in symbolic skills.

Younger children made somewhat greater gains during the counting training than older children in arithmetic and number line scores. This may be due to a difference in time spent at school between the children. The correlation of scores with the age of the children may be indicative of a catch-up effect in younger children, after more instruction. However, the absence of correlations between age and most measures at pretest indicates that this explanation does not sufficiently explain the current results. Alternatively, younger children may have found the activities from the training more appealing, or they may have complied more with instructions set by the trainer, resulting in greater training effects. The effects are contrary to the results presented in the meta-analysis by Kroesbergen and Van Luit (2003), who reported greater training effects of older children. It should be noted, however, that these concerned between-study differences, which may be the result of differences between trainings, and that this is not necessarily indicative of similar within-group moderation effects.

The finding that training gains are moderated by the SES of children (Starkey et al., 2004) could not be replicated. The absence of a moderation effect of SES may be caused by the criterion for group membership. In the current study, children were classified based on the educational level of the parents, while children in the study by Starkey et al. (2004) were classified based on parental income. Although both are indicative of SES, these constructs may have different implications for child development. More specifically, any difference in material resources such as educational materials for children, that may have been associated with differences in training gains in the cited study, may not have been relevant for the groups constructed in the current study. A second cause of the disparity might be the inequality in incomes between families, which is smaller in the Netherlands than in the United States (Central Intelligence Agency, n.d.) and may therefore have smaller consequences for child outcomes.

Future research is needed to elaborate on the parameters of similar training programs. For example, it may be investigated what the effects are of the duration of a training. A meta-analysis concerning the effects of mathematics and number sense trainings has suggested that longer trainings yielded smaller training gains (Kroesbergen and Van Luit, 2003). However, the authors proposed that this was due to differences in scope of the training studies: shorter trainings aimed to improve a more narrow range of skills, leading to more improvement in fewer skills. In a study investigating two training programs with a similar scope, greater training gains and more transfer were reported for the training with the more extensive time span (Toll and Van Luit, 2014). This difference in training gains was significant for general mathematics, and marginally significant for arithmetic. Other evidence concerning the duration of training is scarce, although effects of very short number intervention studies of only four sessions have been reported (Ramani and Siegler, 2008, 2011; Whyte and Bull, 2008).

Also, the range of numbers included is a topic that may be investigated in future research. In the current study, numbers up to 50 were included in the training programs, but other studies have reported on trainings using number ranges up to 10 (Siegler and Ramani, 2008, 2009), up to 15 (Van Luit and Schopman, 2000; Blöte et al., 2006), up to 20 (Fisher et al., 2011) or up to 21 (Baroody et al., 2009). It is likely that children of different ages benefit to a different extent from training programs that focus on different number ranges, and that older children benefit more from broader number ranges as they are already familiar with smaller numbers. However, the exact effect of the inclusion of different number ranges in training programs is as yet unknown.

A limitation of the current study is the sample selection. In the current study, all children were eligible for participation, while not every child was in direct need of a number sense intervention. This may have limited the gains children made during the trainings compared to the control group: children not at-risk for delays in number sense typically make gains in number sense that are sufficient to start formal education without intervention, explaining gains in the control group. Also, longitudinal studies are needed to map the benefits of the interventions fully. Finally, the matching procedure in the current study, in which children were matched at school-level, ensured great variation in number knowledge between children in each training group. Smaller variation in number knowledge may be more beneficial to training gains, because of a more equal level between children at the start of the training, making activities similarly useful to all children in a training group.

A second limitation is the number range covered by the tasks used to evaluate children’s progress in numerical skills. This number range differed per task, with number line tasks ranging up to 100, arithmetic and counting items dealing with quantities up to 20, and comparison tasks only ranging up to 9. This difference in tasks hampers a full comparison in progress between tasks. Conclusions, therefore, can only be made with regard to the comparison in progress between experimental groups and the control group, and not with regard to any difference in progress between various tasks used to index numerical skills. Moreover, number ranges covered during the training sessions only partially overlapped with the pre- and post-tests. Perhaps training gains would be larger if the same number ranges were covered in the training tasks.

Nevertheless, the current study adds to the body of literature by providing experimental evidence for the importance of counting to advance mapping skills and arithmetic skills, and the smaller, non-significant training gains after a number line training. Non-symbolic skills were not influenced by training at all. These findings are of both theoretical and practical significance, because of the implications they have for theories concerning the building of mapping skills and its consequence for arithmetic development, and because of the clear distinction they make in effectiveness of different training activities, which has clear and large implications for the effectiveness of school curricula focusing on number sense.

## Author Contributions

IF-vdB was in charge of the data collection, supervising undergraduate research assistants, and designing the materials, as well as writing the main part of the manuscript. EK checked the data collection decisions and materials, gave advice to improve the materials, and contributed to the manuscript with advice about the theoretical framing of the study as well as suggestions with regard to presentation, and by adding improvements to the text. She wrote major parts of the research proposal based on which the study was funded. JVL contributed to the manuscript with advice about the theoretical framing of the study as well as suggestions with regard to presentation and by adding improvements to the text. He wrote major parts of the research proposal based on which the study was funded.

## Conflict of Interest Statement

The authors declare that the research was conducted in the absence of any commercial or financial relationships that could be construed as a potential conflict of interest.

## Footnotes

1 http://www.pstnet.com

## References

[B1] Association for Universities in the Netherlands (2004). *The Netherlands Code of Conduct for Academic Practice: Principles of Good Academic Teaching and Research.* Available at: https://www.uu.nl/en/files/the-netherlands-code-of-conduct-for-academic-practice-2004-version-2014pdf

[B2] Baroody A. J. Eiland M. Thompson B. (2009). Fostering at-risk preschoolers’ number sense. *Early Educ. Dev.* 20 80–128. 10.1080/10409280802206619

[B3] Barth H. C. Paladino A. M. (2011). The development of numerical estimation: evidence against a representational shift. *Dev. Sci.* 14 125–135. 10.1111/j.1467-7687.2010.00962.x 21159094

[B4] Benjamini Y. Hochberg Y. (1995). Controlling the false discovery rate: a practical and powerful approach to multiple testing. *J. R. Stat. Soc.* 57 289–300. 10.2307/2346101

[B5] Berteletti I. Lucangeli D. Piazza M. Dehaene S. Zorzi M. (2010). Numerical estimation in preschoolers. *Dev. Psychol.* 46 545–551. 10.1037/a0017887 20210512

[B6] Blöte A. W. Lieffering L. M. Ouwehand K. (2006). The development of many-to-one counting in 4-year-old children. *Cogn. Dev.* 21 332–348. 10.1016/j.cogdev.2005.10.002

[B7] Booth J. L. Siegler R. S. (2006). Developmental and individual differences in pure numerical estimation. *Dev. Psychol.* 41 189–201. 10.1037/0012-1649.41.6.189 16420128

[B8] Booth J. L. Siegler R. S. (2008). Numerical magnitude representations influence arithmetic learning. *Child Dev.* 79 1016–1031. 10.1111/j.1467-8624.2008.01173.x 18717904

[B9] Central Intelligence Agency (n.d.). *The World Factbook: Distribution of Family Income – Gini Index (ISSN 1553-8133).* Available at: https://www.cia.gov/library/publications/the-world-factbook/fields/2172.html

[B10] Clarke B. Shinn M. R. (2004). A preliminary investigation into the identification and development of early mathematics curriculum-based measurement. *School Psychol. Rev.* 33 234–248.

[B11] Dackermann T. Fischer U. Huber S. Nuerk H.-C. Moeller K. (2016). Training the equidistant principle of number line spacing. *Cogn. Process.* 17 243–258. 10.1007/s10339-016-0763-8 27075185

[B12] Dehaene S. (1997). *The Number Sense: How the Mind Creates Mathematics.* New York, NY: Oxford University Press.

[B13] Dehaene S. (2001). Précis of the number sense. *Mind Lang.* 16 16–36. 10.1111/1468-0017.00154

[B14] Desoete A. Ceulemans A. De Weerdt F. Pieters S. (2012). Can we predict mathematical learning disabilities from symbolic and non-symbolic comparison tasks in kindergarten? Findings from a longitudinal study. *Br. J. Educ. Psychol.* 82 64–81. 10.1348/2044-8279.002002 21199482

[B15] Desoete A. Praet M. Titeca D. Ceulemans A. (2013). Cognitive phenotype of mathematical learning disabilities: what can we learn from siblings? *Res. Dev. Disabil.* 34 404–412. 10.1016/j.ridd.2012.08.022 23023299

[B16] Dyson N. I. Jordan N. C. Glutting J. (2013). A number sense intervention for low-income kindergartners at risk for mathematics difficulties. *J. Learn. Disabil.* 46 166–181. 10.1177/0022219411410233 21685346 PMC3566272

[B17] Fisher U. Moeller K. Bientzle M. Cress U. Nuerk H.-C. (2011). Sensori-motor spatial training of number magnitude representation. *Psychon. Bull. Rev.* 18 177–183. 10.3758/s13423-010-0031-3 21327351

[B18] Friso-van den Bos I. Kolkman M. E. Kroesbergen E. H. Leseman P. P. M. (2014). Explaining variability: numerical representations in 4- to 8-year old children. *J. Cogn. Dev.* 33 23–29. 10.1080/15248372.2012.742900

[B19] Geary D. C. Hoard M. K. Byrd-Craven J. Nugent L. Numtee C. (2007). Cognitive mechanisms underlying achievement deficits in children with mathematical learning disability. *Child Dev.* 78 1343–1359. 10.1111/j.1467-8624.2007.01069.x 17650142 PMC4439199

[B20] Gersten R. Chard D. J. (1999). Number sense: rethinking arithmetic instruction for students with mathematical disabilities. *J. Spec. Educ.* 33 18–28. 10.1177/002246699903300102

[B21] Gracia-Bafalluy M. Noël M.-P. (2008). Does finger training increase young children’s numerical performance? *Cortex* 44 368–375. 10.1016/j.cortex.2007.08.020 18387567

[B22] Hornung C. Schiltz C. Brunner M. Martin R. (2014). Predicting first-grade mathematics achievement: the contributions of domain-general cognitive abilities, nonverbal number sense, and early number competence. *Front. Psychol.* 5:272. 10.3389/fpsyg.2014.00272 24772098 PMC3983481

[B23] Jordan N. C. Glutting J. Dyson N. I. Hassinger-Das B. Irwin C. (2012). Building kindergartners’ number sense: a randomized controlled study. *J. Exp. Child Psychol.* 104 647–660. 10.1037/a0029018 25866417 PMC4389641

[B24] Kolkman M. E. Kroesbergen E. H. Leseman P. P. M. (2013). Early numerical development and the role of non-symbolic and symbolic skills. *Learn. Instr.* 25 95–103. 10.1016/j.learninstruc.2012.12.001

[B25] Krajewski K. Nieding G. Schneider W. (2008). Kurz- und langfristige Effekte mathematischer Frühförderung im Kindergarten durch das Programm “Mengen, zählen, Zahlen” [Short- and long-term effects of early mathematical support in kindergarten through the programme “Quantities, counting, numbers”]. *Z. Entwicklungspsychol. Pädagog. Psychol.* 40 135–146. 10.1026/0049-8637.40.3.135

[B26] Kroesbergen E. H. Van Luit J. E. H. (2003). Mathematics interventions for children with special educational needs. *Remedial Spec. Educ.* 24 97–114. 10.1177/07419325030240020501

[B27] Kroesbergen E. H. Van ‘t Noordende J. E. Kolkman M. E. (2012). “Number sense in low-performing kindergarten children: effects of a working memory and a number sense training,” in *Reading, Writing, Mathematics and the Developing Brain: Listening to Many Voices*, eds Breznitz Z. Rubinsten O. Molfese V. J. Molfese D. (New York, NY: Springer Publications), 295–313. 10.1007/978-94-007-4086-0_16

[B28] Laski E. V. Siegler R. S. (2007). Is 27 a big number? Correlational and causal connections among numerical categorization, number line estimation, and numerical magnitude comparison. *Child Dev.* 78 1723–1743. 10.1111/j.1467-8624.2007.01087.x 17988317

[B29] Le Corre M. Carey S. (2007). One, two, three, four, nothing more: an investigation of the conceptual sources of the verbal counting principles. *Cognition* 105 395–438. 10.1016/j.cognition.2006.10.005 17208214 PMC3880652

[B30] Lipton J. S. Spelke E. S. (2005). Preschool children’s mapping of number words to nonsymbolic numerosities. *Child Dev.* 76 978–988. 10.1111/j.1467-8624.2005.00891.x 16149996

[B31] Maertens B. De Smedt B. Sasanguie D. Elen J. Reynvoet B. (2016). Enhancing arithmetic in pre-schoolers with comparison or number line estimation training: does it matter? *Learn. Instr.* 46 1–11. 10.1016/j.learninstruc.2016.08.004

[B32] Malofeeva E. Day J. Saco X. Young L. Ciancio D. (2004). Construction and evaluation of a number sense test with head start children. *J. Educ. Psychol.* 96 648–659. 10.1037/0022-0663.96.4.648

[B33] Moeller K. Martignon L. Wessolowski S. Engel J. Nuerk H.-C. (2011). Effects of finger counting on numerical development – The opposing views of neurocognition and mathematics education. *Front. Psychol. Cogn.* 2:328. 10.3389/fpsyg.2011.00328 22144969 PMC3225925

[B34] Mundy E. Gilmore C. K. (2009). Children’s mapping between symbolic and nonsymbolic representations of number. *J. Exp. Child Psychol.* 103 490–502. 10.1016/j.jecp.2009.02.003 19327782

[B35] Noël M.-P. (2005). Finger gnosia: a predictor of numerical abilities in children? *Child Neuropsychol.* 11 413–430. 10.1080/09297040590951550 16306017

[B36] Noël M.-P. Rousselle L. (2011). Developmental changes in the profiles of dyscalculia: an explanation based on a double exact-and-approximate number representation model. *Front. Hum. Neurosci.* 5:165. 10.3389/fnhum.2011.00165 22203797 PMC3243900

[B37] Passolunghi M.-C. Lanfranchi S. (2012). Domain-specific and domain-general precursors of mathematical achievement: a longitudinal study from kindergarten to first grade. *Br. J. Educ. Psychol.* 82 42–63. 10.1111/j.2044-8279.2011.02039.x 22429058

[B38] Piaget J. (1970). *Genetic Epistemology.* New York, NY: Norton.

[B39] Ramani G. B. Siegler R. S. (2008). Promoting broad and stable improvements in low-income children’s numerical knowledge through playing number board games. *Child Dev.* 79 375–394. 10.1111/j.1467-8624.2007.01131.x 18366429

[B40] Ramani G. B. Siegler R. S. (2011). Reducing the gap in numerical knowledge between low- and middle-income preschoolers. *J. Appl. Dev. Psychol.* 32 146–159. 10.1016/j.appdev.2011.02.005 18801120

[B41] Schneider M. Merz S. Stricker J. De Smedt B. Torbeyns J. Verschaffel L. (2018). Associations of number line estimation with mathematical competence: a meta-analysis. *Child Dev.* 10.1111/cdev.13068 [Epub ahead of print]. 29637540

[B42] Siegler R. S. Booth J. L. (2004). Development of numerical estimation in young children. *Child Dev.* 75 428–444. 10.1111/j.1467-8624.2004.00684.x 15056197

[B43] Siegler R. S. Opfer J. E. (2003). The development of numerical estimation: evidence for multiple representations of numerical quantity. *Psychol. Sci.* 14 237–244. 10.1016/j.cogpsych.2004.01.002 12741747

[B44] Siegler R. S. Ramani G. B. (2008). Playing linear numerical board games promotes low-income children’s numerical development. *Dev. Sci.* 11 655–661. 10.1111/j.1467-7687.2008.00714.x 18801120

[B45] Siegler R. S. Ramani G. B. (2009). Playing linear number board games – but not circular ones – improves low-income preschoolers’ numerical understanding. *J. Educ. Psychol.* 101 545–560. 10.1037/a0014239

[B46] Simms V. Muldoon K. Towse J. (2013). Plane thinking: mental representations in number line estimation as a function of orientation, scale, and counting proficiency. *J. Exp. Child Psychol.* 115 468–480. 10.1016/j.jecp.2013.03.011 23688966

[B47] Spelke E. S. (2000). Core knowledge. *Am. Psychol.* 55 1233–1243. 10.1037/0003-066X.55.11.1233 11280937

[B48] Starkey P. Klein A. Wakeley A. (2004). Enhancing young children’s mathematical knowledge through a pre-kindergarten mathematics intervention. *Early Child. Res. Q.* 19 99–120. 10.1016/j.ecresq.2004.01.002

[B49] Toll S. W. M. Van Luit J. E. H. (2014). Effects of remedial numeracy instruction throughout kindergarten starting at different ages: evidence from a large-scale longitudinal study. *Learn. Instr.* 33 39–49. 10.1016/j.learninstruc.2014.03.003

[B50] Van Luit J. E. H. Schopman E. A. M. (2000). Improving early numeracy of young children with special educational needs. *Remedial Spec. Educ.* 21 27–40. 10.1177/074193250002100105

[B51] Van Luit J. E. H. Van de Rijt B. A. M. (2009). *Utrechtse Getalbegrip Toets-Revised [Early Numeracy Test-Revised].* Doetinchem: Graviant.

[B52] Whyte J. C. Bull R. (2008). Number games, magnitude representation, and basic number skills in preschooler. *Dev. Psychol.* 44 588–596. 10.1037/0012-1649.44.2.588 18331146

[B53] Wilson A. J. Revkin S. K. Cohen D. Cohen L. Dehaene S. (2006). An open trial assessment of “The Number Race”, An adaptive computer game for remediation of dyscalculia. *Behav. Brain Funct.* 2:20. 10.1186/1744-9081-2-20 16734906 PMC1523349

[B54] Wong T. T.-Y. Ho C. S. H. Tang J. (2016). The relation between ANS and symbolic arithmetic skills: the mediating role of number-numerosity mappings. *Contemp. Educ. Psychol.* 46 208–217. 10.1016/j.cedpsych.2016.06.003

[B55] Zhang D. Ding Y. Stegall J. Mo L. (2012). The effect of visual-chunking-representation accommodation on geometry testing for students with math disabilities. *Learn. Disabil. Res. Pract.* 27 167–177. 10.1111/j.1540-5826.2012.00364.x 24166208

